# Orthostatic hypotension is associated with malnutrition diagnosed by GLIM in elderly hypertensive patients

**DOI:** 10.1186/s12877-022-03546-x

**Published:** 2022-11-16

**Authors:** Qizhe Zhang, Shanshan Shen, Huilan Guan, Jingmei Zhang, Xujiao Chen

**Affiliations:** 1 Department of Medicine, The Second College of Clinical Medicine, Zhejiang Chinese Medical University, Hangzhou, China; 2 Department of Geriatric, Zhejiang Hospital, 310013 Hangzhou, China

**Keywords:** Orthostatic hypotension, Malnutrition, Muscle mass loss, CGA, Risk factors

## Abstract

**Background:**

Orthostatic Hypotension (OH) and malnutrition, are common health problems in elderly hypertensive patients. This study aimed to analyze the relationship between malnutrition and OH in elderly hypertensive patients.

**Methods:**

This is a cross-sectional single-center study. All participants underwent a Comprehensive Geriatric Assessment (CGA), in which malnutrition was defined according to the Global Leadership Initiative on Malnutrition (GLIM) criteria based on four different methods of diagnosing muscle mass loss. Furthermore, the accuracy of these methods was verified by Receiver Operating Characteristic (ROC) analysis. Univariate and multivariate logistic regression analyses were used to identify risk factors for OH in elderly hypertensive patients.

**Results:**

For GLIM criteria, when Fat-Free Mass Index (FFMI) was the gold standard for muscle mass loss, the Area Under ROC Curve (AUC) values for Upper Arm Circumference (UAC), Calf Circumference (CC), and Hand Grip Strength (HGS) were 0.784, 0.805, and 0.832, with moderate accuracy in diagnosing malnutrition. Multivariate analysis showed that females, Diabetes Mellitus (DM), diuretics, and malnutrition diagnosed by GLIM-UAC were risk factors for OH in elderly hypertensive patients.

**Conclusion:**

Prompt detection of malnutrition in the elderly and attention to changes in UAC may be critical. Similarly, we should strengthen medication and disease management in elderly hypertensive patients.

## Introduction

According to the World Health Organization (WHO), the global population over the age of 60 will exceed 1.2 billion by 2025. The aging process in the elderly often leads to a degradation of the function of the blood pressure regulatory system, which results in a significant increase in the incidence of Orthostatic Hypotension (OH) [1, 2]. OH, is defined as a clinical manifestation of a 20 mmHg drop in systolic blood pressure or a 10 mmHg drop in diastolic blood pressure [3]. Available data suggest that the potential prevalence of OH in older adults over 65 years is generally 30%[4], and another study showed that about 84.4% of elderly hospitalized patients over the age of 75 suffered from OH [5]. The most common comorbidity of OH is Hypertension (HTN) [6]. The presence of OH can complicate the management of patients with HTN, as treating one may worsen the other [7]. In addition, older adults with HTN and OH had a more than a two-fold increased risk of falls [8]. Therefore, OH in elderly hypertensive patients should be more concerned.

Moreover, malnutrition is an important geriatric syndrome, which refers to insufficient, excessive, or abnormal nutrient intake, resulting in incompatibility with the body’s nutritional needs, adversely affecting the body’s morphological functions, and ultimately leading to adverse events such as sarcopenia and frailty [9–13]. In addition, the imbalance and gait variability caused by the decrease in muscle mass and muscle strength associated with sarcopenia can increase the probability of falls in the elderly [14]. Epidemiological studies have shown that the prevalence of malnutrition in hospitalized elderly is 30–61% [15]. So the diagnosis of malnutrition is crucial. To date, although there is no consensus on the assessment of malnutrition, various screening tools have been developed. The Global Leadership Initiative on Malnutrition (GLIM), the latest consensus, is a two-step diagnostic process. GLIM is widely used in many studies, and one of the points of contention is how to define muscle mass loss. Although dual-energy X-ray absorptiometry (DXA) and Bioelectrical Impedance Analysis (BIA) are recommended for measuring muscle mass, they have the disadvantage of being costly and time-consuming [16]. The newly proposed method of assessing lower extremity muscle thickness by ultrasound to diagnose muscle mass is equally complex [17]. Therefore, physical examination or anthropological measures, including Upper Arm Circumference (UAC), Calf Circumference (CC), and Hand Grip Strength (HGS) are often used for estimation, but their accuracy remains to be verified.

Malnutrition and malnutrition risk were risk factors for OH in a study using a Mini-Nutrition Assessment (MNA) as a diagnostic method [18]. And it is thought that this may be due to the nutritional-related loss of muscle mass and the effect of muscle tone on vascular and autonomic nerve function, leading to OH. However, this was only a study conducted in outpatient older adults, and we conducted this study to examine the relationship between OH and malnutrition in hospitalized older adults with HTN. In this article, we will focus on malnutrition and OH prevalence, verify the accuracy of the different methods measuring muscle mass loss, and analyze the influencing factors of OH in hypertensive elderly patients to provide a theoretical basis for subsequent precise prevention and interventions.

## Methods

### Design, setting and sample

Our observational study was conducted at the Zhejiang Hospital, Hangzhou, China, from 15 to 2015 to 18 February 2020. According to the Declaration of Helsinki, each subject provided written informed consent before conducting this study.

### Variables and measurements

All patients completed the Comprehensive Geriatric Assessment (CGA), which involved an investigation of disease and medication history. Disease investigations included OH, HTN, Coronary Heart Disease (CHD), Chronic Obstructive Pulmonary Disease (COPD), and Diabetes Mellitus (DM). Drug investigations included Angiotensin-Converting Enzyme Inhibitor (ACEI), Angiotensin Receptor Blocker (ARB), Beta-Blocker (BB), Calcium Channel Blocker (CCB), Psychotropic, and statins. At the same time, questionnaires including Mini-Mental State Examination (MMSE), Instrumental Activities of Daily Living (IADL), and 15-item Geriatric Depression Scale (GDS-15) were completed.

The diagnosis of OH and HTN was conducted based on blood pressure measurements. We diagnosed OH with a transition from supine to standing, a 20 mmHg drop in systolic blood pressure, and a 10 mmHg drop in diastolic blood pressure within three minutes of the test [19]. And the diagnosis of HTN was defined according to relevant guidelines as systolic blood pressure ≥ 140 and/or diastolic blood pressure ≥ 90 mmHg, after repeating the measurement multiple times on different days [20].

Our study used the latest consensus, GLIM, to diagnose malnutrition. GLIM is based on MNA-SF [21] to complete a two-step process that includes a clinical phenotype, weight loss, low Body Mass Index(BMI) and low muscle mass loss; and an etiological phenotype, reduced food intake or absorption, inflammation, or disease burden.

Because of the complexity of indicators to monitor the inflammatory state, it was not considered in this study. And disease burden referred to most chronic organ diseases, including heart failure, COPD, chronic kidney or liver disease, and cancer [22]. Muscle mass loss took four different diagnostic forms. Subjects received a BIA measurement ( Inbody S10) to record Fat-Free Mass Index (FFMI) values and used a grip dynamometer to measure the dominant hand three times to select the maximum value. Based on resistance and reactance at 50 kHz, Fat-Free Mass (FFM) was calculated according to the Geneva equation. Subsequently, FFMI was calculated as FFM/height^2^ [23]. CC and UAC were measured with a tape measure while the participant was standing. Tape around the thickest part of the arm biceps to get UAC, and circle around the thickest part of the calf to get CC (Table 1).

**Table 1:** Cut off values for the phenotype and etiologic criteria of GLIM

GLIM criteria	
Phenotype criteria	**Weight loss**: >5%(six months)
	**Low BMI**: < 18.5 kg/m^2^(< 70years) or < 20 kg/m^2^ (≥ 70years)
	**Muscle mass loss**: Low FFMI or low UAC or low CC or low HGS Low FFMI: male<17 kg/m^2^;female<15 kg/m^2^ Low UAC: male<33 cm;female<26 cm Low CC: male<34 cm;female<33 cm Low HGS: male<27 kg;female<16 kg
Etiology criteria	**Reduced food intake**: <5% of energy requirement or any reduction for two weeks
	**Disease burden**: Heart failure or chronic obstructive pulmonary disease or rheumatoid arthritis or chronic kidney or liver disease or cancer

### Statistical analysis

Data analysis was completed using IBM SPSS software Version 26.0 for Windows. Quantitative variables were expressed as mean ± standard deviation. Quantitative variable distributions were assessed using the Kolmogorov-Smirnov test. Differences between quantitative variables were analyzed using Student’s t-test and nonparametric tests (Mann-Whitney) for variables that did not follow a normal distribution. Receiver operating characteristic (ROC) curves were estimated for each muscle mass loss diagnosed method of GLIM. The area under the curve of ROC (AUC) was used to estimate the discriminative ability. The indications for the diagnostic values were as follows: 0.5, none; 0.5 to 0.7, poor; 0.7 to 0.9, moderate; and 0.9 to 1, 2, good. We designed a multivariate logistic regression model. The dependent variable was OH. A multiple logistic regression model included factors identified as potentially significant (p < 0.05) in univariate analysis. A P-value of < 0.05 was considered statistically significant.

## Results

In the datasets, we enrolled 532 patients, 157 patients were excluded because of incomplete clinical data (n = 132), active tumor (n = 10), and clinical diagnosis of acute heart, liver, and kidney injury (n = 15). The final study population consisted of 375 patients.

### Characteristics of the subgroup based on OH

Table 2 shows the characteristics of the total study population. Of the participants, the median age was 86 years, and 55.20% were males. More than half participants were dentures (65.55%) and living alone (59.47%). Patients were divided into two groups according to the presence of OH. Malnutrition diagnosed by GLIM-UAC and GLIM-HGS, the proportion of males and the use of diuretics were statistically significantly different between the OH-positive and OH-negative groups.

**Table 2:** General characteristics

Parameter	Total n = 375	OH(+) n = 68	OH(-) n = 307	P-value
**Demographic characteristics**				
Age, y	86(78,91)	88(78,92)	86(77,91)	0.120
Male, n (%)	207(55.20)	48(70.59)	159(51.79)	0.005*
Smoking, n (%)	108(28.80)	26(38.24)	82(26.71)	0.058
Drinking, n (%)	110(31.73)	22(32.35)	97(31.60)	0.903
Denture, n (%)	239(63.73)	49(72.06)	190(61.89)	0.115
BMI, kg/m^2^	24(21,27)	24(22,26)	24(21,27)	0.361
Living alone, n (%)	223(59.47)	39(57.32)	184(59.93)	0.695
**Commodities**, n (%)				
DM	300(80.00)	48(70.59)	252(82.08)	0.032
CHD	22(5.87)	26(38.24)	92(29.97)	0.184
COPD	22(5.87)	7(10.29)	15(4.89)	0.086
**CGA**, n (%)				
MMSE	25(20,28)	26(23,28)	25(20,28)	0.102.
ADL	100(95,100)	100(95,100)	100(90,100)	0.775
IADL	6(4,8)	6.5(5,8)	6(4,8)	0.552
ICI	0(0,4)	0(0,3)	0(0,4)	0.889
PSQI	7(3,11)	7.5(4,10.75)	7(3,11)	0.460
GDS-15	2(1,5)	2(0,4.75)	2(1,5)	0.313
**Malnutrition**, n (%)				
GLIM-FFMI	141(37.60)	19(27.94)	122(39.74)	0.069
GLIM-UAC	93(24.80)	8(11.76)	85(27.69)	0.013*
GLIM-CC	102(27.20)	12(17.65)	90(29.32)	0.050
GLIM-HGS	121(32.27)	15(22.06)	106(34.53)	0.047*
**Medication**, n (%)				
ACEI/ARB	128(34.13)	25(36.76)	103(33.55)	0.601
BB	81(21.60)	15(22.06)	66(21.50)	0.919
CCB	178(46.67)	33(48.53)	145(47.23)	0.846
Diuretics	216(57.60)	25(30.88)	191(62.21)	0.000*
Psychotropic	71(18.93)	15(22.06)	56(18.24)	0.467
statins	181(48.27)	35(51.47)	146(47.56)	0.559

### Nutritional screening and evaluation results

**Fig. 1:**
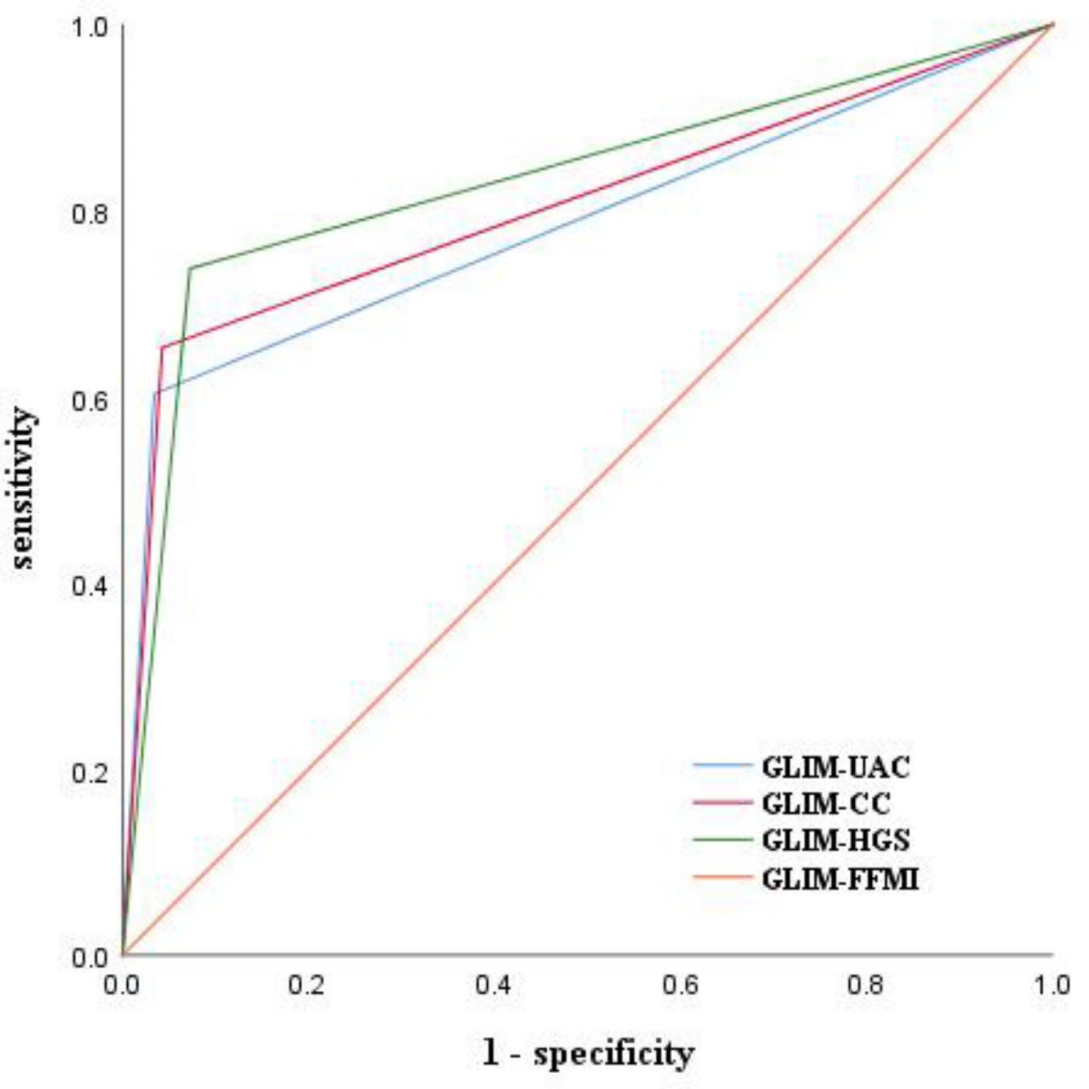
Receiver operating characteristic (ROC) curve for prediction of malnutrition by glim, the diagnosis of muscle mass is based on the FFMI, CC, UAC, and HGS.

The incidence of malnutrition risk with MNA-SF ≤ 11 points was 42.67%. And the overall malnutrition rate was 45.07%. Patients who met each criterion met the malnutrition status are shown in Table 3. In addition, the GLIM defined in this study set different criteria for muscle mass loss, and AUC calculated by using FFMI-based diagnosis of malnutrition as the gold standard indicated that all three screening tools had a moderate effect (AUC of 0.784, 0.805, and 0.832 for UAC, CC, and HGS) (Fig. 1).

**Table 3:** The prevalence of patients meeting each criterion of the GLIM framework

GLIM criteria	Prevalence, n (%)
**GLIM phenotypic criteria**	
Weight loss	94(25.07)
Low BMI	102(27.20)
muscle mass loss FFMI CC UAC HGS	75(20.00) 106(28.27) 148(39.47) 71(18.93)
**GLIM etiologic criteria**	
Reduce food intake	72(19.20)
Disease burden	94(25.07)

### Analysis of the influencing factors of OH in hypertensive patients

After controlling for potential confounders, univariate and multivariate analyses to assess risk and protective factors for OH in elderly hypertensive patients. OH was associated with gender, DM, and GLIM-UAC in hypertensive patients. Male was its protective factor (Odds Ratio (OR) 0.433; 95% Confidence Interval (CI) 0.241–0.780; p = 0.005). DM, diuretics and GLIM-UAC were risk factors (OR, 1.895; 95%CI, 1.017–3.534; p = 0.044), (OR, 2.667; 95%CI, 1.517–4.688; p = 0.001), (OR, 2.375; 95%CI, 1.066–5.293; p = 0.034) (Table 4).

**Table 4:** Univariate and multivariate analyses assess risk factors for patients with OH

Parameter	Univariate analysis			Multivariate analysis		
	OR	95% CI	p-value	OR	95% CI	p-value
Age	1.023	0.990–1.057	0.176			
Male	0.448	0.254–0.790	0.006*	0.433	0.241–0.780	0.005*
BMI	1.009	0.956–1.006	0.742			
Smoking	0.589	0.339–1.021	0.059			
Drinking	0.966	0.550–1.694	0.903			
DM	1.909	1.050–3.470	0.034*	1.895	1.017–3.534	0.044*
CHD	0.691	0.400-1.194	0.186			
COPD	0.448	0.175–1.144	0.093			
ADL	1.014	0.990–1.038	0.251			
IADL	1.060	0.948–1.185	0.309			
MMSE	1.053	1.000-1.108	0.050			
PSQI	1.014	0.959–1.073	0.618			
ACEI/ARB	0.868	0.503–1.501	0.613			
BB	0.968	0.513–1.825	0.919			
CCB	0.949	0.561–1.606	0.846			
Diuretics	2.832	1.643–4.881	0.000*	2.667	1.517–4.688	0.001*
Psychotropic	0.788	0.415–1.498	0.468			
Stains	0.855	0.505–1.446	0.559			
GLIM-FFMI	1.701	0.955–3.028	0.071			
GLIM-UAC	2.872	1.318–6.258	0.008*	2.375	1.066–5.293	0.034*
GLIM-CC	1.935	0.990–3.783	0.053			
GLIM-HGS	1.837	0.988–3.414	0.055			

## Discussion

To our knowledge, the results of our research showed that the prevalence of malnutrition and OH were 45.07% and 18.13%. CC, UAC, and HGS all three screening tools had a moderate effect in diagnosing malnutrition. It also revealed that malnutrition diagnosed by GLIM-UAC, DM, female and long-term use of diuretics were risk factors for OH in hypertensive elderly patients.

Our study used the latest consensus, GLIM, to diagnose malnutrition. This is an easy way for clinicians to apply the available tools and methods [22]. In our sample, the results of the three muscle mass loss diagnosing tools, HGS, CC, and UAC, were validated to have a moderate accuracy in diagnosing malnutrition, using FFMI as the gold standard. Sanchez-Rodriguez D used the same methodology as ours to reach similar conclusions. But they were in the community, and we were in the elderly who were hospitalized [17]. Similarly, a survey in Spain yielded similar results [24]. Therefore, we believe that measurements of CC, UAC, and HGS can be used in place of FFMI in limited settings such as primary hospitals or communities, and recommended to first refine the measurement of CC, HGS, and UAC in high-risk populations.

Previous studies have reported that both malnutrition and malnutrition risk were associated with OH [18, 25]. Our findings suggested that malnutrition diagnosed by GLIM-UAC is a risk factor for OH in elderly hypertensive patients. However, this was not observed in several other diagnoses such as GLIM-CC, which may be the reason for the presence of invisible lower extremity edema in the elderly, affecting the results of CC and BIA measurements. Vitamins as one the important nutrients, numerous studies have found that vitamin D plays an important role in OH [26], and this conclusion has been verified in both men and women [27, 28]. It is considered that vitamin D has a role in blood pressure control and intravascular volume, vitamin D deficiency may promote OH through this mechanism. Vitamin D affects the vasopressor response by down regulating the renin-angiotensin-aldosterone system and may be involved in regulating the vascular response in the upright state. At the same time, vitamin D metabolites modulate the gene expression of neurotrophic factors, resulting in decreased compensatory mechanisms during standing [29]. However, studies conducted in older Irish communities contradicted our conclusions [26, 30]. Because malnutrition is preventable, nutritional status should be examined when assessing changes in blood pressure, especially in elderly hypertensive patients. Meanwhile, as another important nutrient, about 50%-75% of protein is stored in skeletal muscle [31]. Protein intake is a master regulator of muscle protein metabolism, affecting the regulation of the dynamic and transient balance between muscle protein synthesis (MPS) and muscle protein breakdown (MPB). Inadequate protein intake can lead to sarcopenia and frailty through multiple mechanisms [32]. And in present, the correlation between OH and muscle mass reduction has been confirmed by multiple studies [33–35].

Our findings proposed that long-term diuretic use and DM were risk factors for OH. Diuretics increase natriuresis and lead to a decrease in urine output, especially in the elderly. Loop diuretics also increase venous volume, which reduces venous return and cardiac output. Several studies reported a significant association of diuretics with OH [36, 37]. Therefore, drug screening is recommended as a first-line approach to OH’s diagnostic and therapeutic workup. It should be aimed at evaluating their indications and benefits to assess discontinuation or dose reduction. With DM being a common condition, DM-related autonomic dysfunction is often considered to be the main mechanism leading to delayed blood pressure recovery as it impairs the pressure reflex-mediated response to blood pressure recovery from hypotension. Similarly, it has been shown that DM patients are significantly more likely to experience OH after thirty seconds of standing than those without DM [38].

This study has certain clinical significance. Considering the risk factors derived from the results, medication and disease management should be included in the actual health management of older adults, especially those with HTN, DM and chronic diuretic use. In addition, nutritional screening should be strengthened, and early detection and diagnosis of malnutrition may facilitate the detection of OH. However, there were some limitations in the present study. First, our study is a cross-sectional, single-center study, and observed associations could not establish a causal nexus between malnutrition and OH. And next, the specific types of diuretics and the intake amount of nutrients such as vitamins and proteins were not analyzed and compared. Further prospective studies are needed to explore the relationship between OH and malnutrition in elderly hypertensive hospitalized patients.

## Conclusion

This study demonstrated that CC, UAC, and HGS have moderate to moderate accuracy in diagnosing malnutrition in elderly hypertensive populations. We also found malnutrition diagnosed by GLIM-UAC, females, DM, and long-term diuretic use were risk factors for OH in elderly hypertensive patients. Therefore, management of disease, medication and nutritional status is necessary.

## Data Availability

The data that support the findings of this study are available from the corresponding author upon reasonable request.

## Abbreviations

FFMI: fat-free mass index
UAC: upper arm circumference
CC: calf circumference
HGS: hand grip strength
BMI: body mass index
OH: orthostatic hypotension
CHD: coronary heart disease
COPD: chronic obstructive pulmonary disease
DM: diabetes mellitus
MMSE: mini-mental state examination
IADL: instrumental activity of daily living
ADL: activity of daily living
GDS-15: 15-item geriatric depression scale
ICI: international consultation on incontinence question
CGA: comprehensive geriatric assessment
ACEI: angiotensin-converting enzyme inhibitor
ARB: angiotensin receptor blocker
BB: beta-blocker
CCB: calcium channel blocker
PSQI: pittsburgh sleep quality index
HTN: hypertension
GLIM: global leadership initiative on malnutrition
DXA: dual-energy x-ray absorptiometry
BIA: bioelectrical impedance analysis
MNA: mini-nutrition assessment
MNA-SF: short-form mini-nutrition assessment
OR: Odds ratio
